# Mathematical Analysis of the ***Escherichia coli*** Chemotaxis Signalling Pathway

**DOI:** 10.1007/s11538-018-0400-z

**Published:** 2018-02-05

**Authors:** Matthew P. Edgington, Marcus J. Tindall

**Affiliations:** 1 Department of Mathematics and Statistics, University of Reading, Whiteknights, PO Box 220, Reading, RG6 6AX UK; 2 Present Address: The Pirbright Institute, Ash Road, Woking, Surrey GU24 0NF UK; 3 Institute for Cardiovascular and Metabolic Research, University of Reading, Whiteknights, PO Box 218, Reading, RG6 6AA UK

**Keywords:** Bacterial chemotaxis, Signalling pathway, Adaptation, Equilibrium, Stability analysis, Overshoot

## Abstract

We undertake a detailed mathematical analysis of a recent nonlinear ordinary differential equation (ODE) model describing the chemotactic signalling cascade within an *Escherichia coli* cell. The model includes a detailed description of the cell signalling cascade and an average approximation of the receptor activity. A steady-state stability analysis reveals the system exhibits one positive real steady state which is shown to be asymptotically stable. Given the occurrence of a negative feedback between phosphorylated CheB (CheB-P) and the receptor state, we ask under what conditions the system may exhibit oscillatory-type behaviour. A detailed analysis of parameter space reveals that whilst variation in kinetic rate parameters within known biological limits is unlikely to lead to such behaviour, changes in the total concentration of the signalling proteins do. We postulate that experimentally observed overshoot behaviour can actually be described by damped oscillatory dynamics and consider the relationship between overshoot amplitude, total cell protein concentration and the magnitude of the external ligand stimulus. Model reductions in the full ODE model allow us to understand the link between phosphorylation events and the negative feedback between CheB-P and receptor methylation, as well as elucidate why some mathematical models exhibit overshoot and others do not. Our paper closes by discussing intercell variability of total protein concentration as a means of ensuring the overall survival of a population as cells are subjected to different environments.

## Introduction

Numerous bacterial species use chemotaxis in order to move through their environment in search of chemoattractants—substances beneficial to their survival. *Escherichia coli* cells use four to six helical flagella in order to create a run and tumble swimming pattern akin to a random walk (Berg and Brown 1972). These flagella are each controlled by membrane-bound motors that cause them to rotate in either counterclockwise (CCW) or clockwise (CW) directions (Porter et al. 2011). CCW rotation causes the flagella to bundle together propelling the cell forward for a period of time (the run). In contrast, CW rotation leads to the flagella flailing apart resulting in random reorientation of the cell (the tumble). The ratio of time spent in runs compared to tumbles is known as the rotational bias and is modulated by the cells’ response to chemicals in the surrounding environment. This allows cells to bias their movement towards CCW flagella rotation (increasing the run length) upon sensing an increased chemoattractant concentration (Berg 2011).

**Fig. 1:**
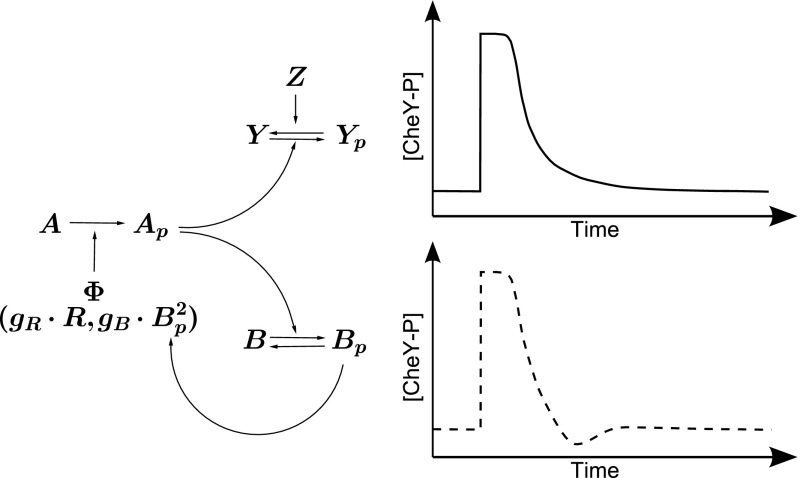
Schematic representation of the intracellular signalling pathway in *E. coli* chemotactic cells (left). Receptors at the cell pole sense an external attractant concentration, determining a receptor activity level ($\varPhi$). At a rate dependent on this activity, CheA autophosphorylates, forming CheA-P. Phosphoryl groups are then passed to either CheY or CheB (giving CheY-P and CheB-P). CheY-P and CheB-P both dephosphorylate. CheZ acts to speed up dephosphorylation of CheY-P. CheY-P is used to control the swimming behaviour of the cell. CheB and CheR are the adaptation components of the chemotaxis pathway. CheB-P alters the receptor state by demethylating receptors, thereby negatively regulating autophosphorylation. Meanwhile, CheR constantly methylates receptors, positively regulating autophosphorylation. The balance of these processes is able to reset receptors to their pre-stimulus state. (Right) Examples of the chemotactic response. The initial rapid response is followed by a period of smooth transient behaviour in which the cell returns to pre-stimulus levels. The upper figure shows a cell response without oscillatory behaviour, whilst the lower demonstrates an oscillatory response

The chemotactic response is the result of a well-characterised intracellular signalling pathway (Wadhams and Armitage 2004), as shown in Fig. 1. *E. coli* cells sense their environment using transmembrane chemoreceptors that each have the ability to sense different extracellular factors (Grebe and Stock 2008). The chemoreceptors are linked to the flagella driving motors of the cell via an intracellular signalling pathway. Chemoreceptors associate with a linker protein CheW and a histidine kinase CheA. In the absence of an attractant gradient, CheA autophosphorylates at a steady rate, forming CheA-P (Wadhams and Armitage 2004). Phosphoryl groups are then transferred from CheA-P onto either a methylesterase CheB or the response regulator CheY. Phosphorylated CheY (CheY-P) may then diffuse within the cytoplasm of the cell to the flagellar motors (Bren et al. 1996; Lipkow et al. 2005). Once at a flagellar motor CheY-P is able to bind the motor switching protein FliM causing an increase in the tumble bias of the cell (Welch et al. 1993). In addition to spontaneous dephosphorylation of CheY-P, a phosphatase CheZ acts to increase the rate at which this dephosphorylation occurs (Likpow 2006). It has been observed that using this signalling pathway *E. coli* cells are able to remain sensitive to over five orders of magnitude in external ligand concentration (Mesibov et al. 1973). This is due to CheR and CheB, the adaptation components of the chemotaxis pathway, which act to reset the chemoreceptors to their pre-stimulus state. CheR constantly methylates the chemoreceptors, increasing their activity (Springer and Koshland 1977), whereas CheB-P demethylates them, decreasing their activity (Stock and Koshland 1978).

Upon sensing a positive chemoattractant gradient, the rate of CheA autophosphorylation is reduced, leading to a reduction in levels of both CheB-P and CheY-P. The reduction in CheY-P levels results in less binding to FliM causing the flagellar motors to experience a greater CCW bias, thereby inducing an extended run. An associated reduction in CheB-P allows chemoreceptors to become methylated due to the action of CheR. This results in a return to the pre-stimulus CheA autophosphorylation rate and in turn CheB-P, CheY-P and flagellar rotation bias all return to their pre-stimulus values.

During responses of this type, *E. coli* chemotactic cells have been known to exhibit a phenomenon known as overshoot. This occurs when, following the response to an external stimulus, the cell exceeds its pre-stimulus value for a transient period of time before returning to it (Fig. 1). Overshoot was first observed experimentally in the cellular response of *E. coli* cells to impulse stimuli, i.e. when a stimulus persists for a very short period of time (Berg and Tedesco 1975; Block et al. 1983; Segall et al. 1986). More recently, it has been shown to exist in response to step changes in external ligand concentrations (Min et al. 2012). Within the theoretical literature, a number of different causes have been postulated. In response to an impulse stimulus, overshoot was shown to increase with the catalytic rate of CheR (Goldman et al. 2009). Methylation crosstalk between different receptor types was also proposed as a possible mechanism producing overshoot (Hansen et al. 2010; Lan et al. 2011). Specifically, non-ligand binding receptors may become methylated due to coupling with ligand binding receptors. For example, in response to aspartate stimuli Tsr (serine-sensing chemoreceptor) receptors may be methylated due to their coupling with Tar (aspartate-sensing chemoreceptor) receptors.

Understanding the response of *E. coli* cells to external attractants has been the subject of experimental work and mathematical models for nearly 40 years. The use of experimental work in informing mathematical model formulation and revision, and likewise the use of models in elucidating cell behaviour, has led to a range of mathematical models being formulated. Many such models (Xin and Othmer 2012; Spiro et al. 1997; Barkai and Leibler 1997) have been formulated and developed to provide a comprehensive description of the cellular processes and include details of receptor methylation, ligand-receptor binding and its subsequent effect on the biochemical signalling cascade, along with a description of motor driving CheY/CheY-P levels. However, including such detail has often led to very complex mathematical models consisting of tens of governing differential equations, making mathematical analysis of the underlying cellular response difficult, if not in many cases, impossible.

The recent model of Clausznitzer et al. (2010) has sought to provide a comprehensive description of the *E. coli* response, by coupling a simplified statistical mechanical description of receptor methylation and ligand binding, with the signalling cascade dynamics. By taking an average approach to the receptor cluster response and exploiting the large separation in timescales of ligand-receptor binding and receptor conformational change (order of milliseconds) versus that of the cell signalling cascade response (seconds to tens of seconds), the authors are able to formulate a model consisting of five nonlinear ordinary differential equations (ODEs). The model is parameterised using data from the literature and is shown to be in good agreement with experimental findings. The size and ability of the Clausznitzer et al. (2010) model to capture the dynamical *E. coli* response, means it is ripe for investigating the role of cellular signalling mechanisms in driving the cellular response. However, this fifth-order nonlinear ODE model is still difficult to treat analytically.

In this work, we undertake a comprehensive mathematical analysis of a number of simplified forms of the model due to Clausznitzer et al. (2010) to elucidate the role of specific aspects of the signalling cascade on the cellular response. We test the hypothesis that the respective system will exhibit oscillatory-type behaviour given the occurrence of a negative feedback between CheB-P and receptors on the cell surface and ask under what conditions this may be exhibited. Having demonstrated oscillations may occur, and determined under what conditions they do, we seek to place these findings in the context of the overall system dynamics and experimental observations regarding overshoot.

We consider a fourth-order reduction of the Clausznitzer et al. (2010) model used in previous theoretical literature. This model, its non-dimensionalisation and parameterisation are presented in Sect. 2. We begin our analysis by conducting a steady-state stability analysis of the governing system of ODEs as detailed in Sect. 3. This is followed in Sect. 4 by analysis of the negative feedback between CheB-P and receptor methylation levels. Section 5 discusses how the theoretical results presented here relate to those in the experimental literature. In Sect. 6, we undertake a series of model reductions to understand the role of the feedback and the effect of timescale separation on the bacterial response as well as outcomes from models previously published in the literature. We conclude in Sect. 7 with a discussion of our results in the context of experimental data regarding variation in the chemotactic response.

## A Mathematical Model of ***E. coli*** Chemotaxis

The mathematical model of *E. coli* signalling due to Clausznitzer et al. (2010) consists of an ODE description of the key signalling chemotactic pathway coupled with that of a statistical mechanical description of ligand-receptor binding. The chemotactic pathway dynamics are described by

1 $$\begin{aligned} \frac{\hbox {d}[A_p]}{\hbox {d}t}= & {} \varPhi k_1([A_T]-[A_p])\!-\!k_2[A_p]([Y_T]\!-[Y_p])- k_3[A_p]([B_T]\!-\![B_p]), \end{aligned}$$

2 $$\begin{aligned} \frac{\hbox {d}[Y_p]}{\hbox {d}t}= & {} k_2 [A_p]([Y_T]-[Y_p])-k_A([Z_T]-[Y_pZ])[Y_p] + k_D[Y_pZ], \end{aligned}$$

3 $$\begin{aligned} \frac{\hbox {d}[Y_pZ]}{\hbox {d}t}= & {} k_A([Z_T]-[Y_pZ])[Y_p]-(k_D+k_Y)[Y_pZ], \end{aligned}$$

4 $$\begin{aligned} \frac{\hbox {d}[B_P]}{\hbox {d}t}= & {} k_3[A_p]([B_T]-[B_p])-k_5[B_p], \end{aligned}$$

within which $k_i$ ($i=1, 2, 3, 5, A, D, Y$) indicate the kinetic rates of each reaction and $[{\ldots }]$ denote the concentration of the appropriate protein with subscripts *T* and *p* indicating the total and phosphorylated concentrations, respectively.

In this study, we consider a reduced form of the model in which it is assumed that association, dissociation and dephosphorylation reactions involving CheY-P and CheZ complexes occur rapidly enough such that the formation of the intermediary complex is ignored, a common assumption in this system when considering the effect of CheZ dephosphorphylation on CheY-P (e.g. Bray et al. 1993; Kollmann et al. 2005). As such, CheZ is assumed to have a constant dephosphorylation effect on CheY-P leading to the reduced fourth-order system

5 $$\begin{aligned} \frac{\hbox {d}[A_p]}{\hbox {d}t}= & {} \varPhi k_1([A_T]-[A_p])-k_2[A_p]([Y_T]-[Y_p])- k_3[A_p]([B_T]-[B_p]), \end{aligned}$$

6 $$\begin{aligned} \frac{\hbox {d}[Y_p]}{\hbox {d}t}= & {} k_2 [A_p]([Y_T]-[Y_p])-k_4[Y_p][Z_T]-k_6[Y_p], \end{aligned}$$

7 $$\begin{aligned} \frac{\hbox {d}[B_P]}{\hbox {d}t}= & {} k_3[A_p]([B_T]-[B_p])-k_5[B_p], \end{aligned}$$

where as before $k_i$ ($i=1, 2, 3, 4, 5, 6$) indicate the kinetic rates of each reaction (Table 1 for details) and $[{\ldots }]$ the concentration of a given protein . Such a reduced form of Clausznitzer et al. (2010) model has been used previously in the theoretical literature (see for example Edgington and Tindall 2015).

The kinetics of receptor methylation are described by

8 $$\begin{aligned} \frac{\hbox {d}m}{\hbox {d}t} = g_R[R_T](1-\varPhi )-g_B[B_p]^2\varPhi , \end{aligned}$$

where *m* is the average methylation level of a receptor within the signalling team, whilst $g_R$ and $g_B$ denote the kinetic rates of receptor methylation by CheR and demethylation by CheB-P, respectively. The receptor signalling team activity ($\varPhi$) in Eqs. (5) and (8) is determined by a Monod–Wyman–Changeux (MWC) description of receptor clustering (Monod et al. 1965)

9 $$\begin{aligned} \varPhi =\frac{1}{1+e^F}, \end{aligned}$$

in which *F* is the free energy of a receptor signalling team and is defined as

10 $$\begin{aligned} F=N\left[ 1-\frac{m}{2}+\ln \left( \frac{1+[L]/K_a^\mathrm{off}}{1+[L]/K_a^\mathrm{on}}\right) \right] . \end{aligned}$$

Here, *N* is the number of chemoreceptors in the signalling team, $1-m/2$ represents the “offset energy” (i.e. the contribution to *F* from addition/removal of one methyl group) and the remainder of the expression is the free energy of an individual Tar receptor with ligand dissociation constants $K_a^{on/off}$ for active/inactive receptors, and [*L*] represents the extracellular ligand concentration.

In contrast to Clausznitzer et al. (2010), we ignore the low-affinity binding of MeAsp to Tsr receptors as per Mello and Tu (2007). For small and intermediate extracellular MeAsp concentrations, this does not significantly alter the dynamical response of the model.

### Parameterisation

The model is informed with the parameter values originally stated in Clausznitzer et al. (2010) as detailed in Table 1. This mathematical model has been shown to be a good fit to experimental data in spite of the low copy numbers associated with CheR and CheB (Clausznitzer et al. 2010). With this being the case, we do not anticipate stochastic effects altering any conclusions drawn from this study.

**Table 1:** Dimensional model parameter values and their respective sources

Symbol	Definition	Value	Source
$[A_T]$	Total concentration of CheA	7.9 $\upmu \hbox {M}$	Li and Hazelbauer (2004)$^\mathrm{a}$
$[B_T]$	Total concentration of CheB	0.28 $\upmu \hbox {M}$	Li and Hazelbauer (2004)$^\mathrm{a}$
$[R_T]$	Total concentration of CheR	0.16 $\upmu \hbox {M}$	Li and Hazelbauer (2004)$^\mathrm{a}$
$[Y_T]$	Total concentration of CheY	9.7 $\upmu \hbox {M}$	Li and Hazelbauer (2004)$^\mathrm{a}$
$[Z_T]$	Total concentration of CheZ	3.8 $\upmu \hbox {M}$	Li and Hazelbauer (2004)$^\mathrm{a}$
$k_1$	CheA autophosphorylation	34 $\hbox {s}^{-1}$	Francis et al. (2002)
$k_2$	Phosphotransfer to CheY	100 $\upmu \hbox {M}^{-1}\hbox {s}^{-1}$	Stewart et al. (2000)
$k_3$	Phosphotransfer to CheB	15 $\upmu \hbox {M}^{-1}\hbox {s}^{-1}$	Stewart et al. (2000)
$k_4$	CheY-P dephosphorylation by CheZ	1.6 $\upmu \hbox {M}^{-1}\hbox {s}^{-1}$	Li and Hazelbauer (2004)
$k_5$	Dephosphorylation of CheB-P	0.7 s$^{-1}$	Stewart et al. (1990)
$k_6$	Dephosphorylation of CheY-P	0.085 s$^{-1}$	Smith et al. (2003)
$g_R$	Methylation by CheR	0.0375 $\upmu \hbox {M}^{-1}\hbox {s}^{-1}$	Clausznitzer et al. (2010)
$g_B$	Demethylation by CheB-P	3.14 $\upmu \hbox {M}^{-2}\hbox {s}^{-1}$	Clausznitzer et al. (2010)
*N*	Number of Tar receptors in a signalling team	18	Endres et al. (2008)
$K_a^\mathrm{on}$	Dissociation constant of an active Tar receptor	0.5 mM	Keymer et al. (2006)
$K_a^\mathrm{off}$	Dissociation constant of an inactive Tar receptor	0.02 mM	Keymer et al. (2006)

$^\mathrm{a}$Calculated from experimental values in Li and Hazelbauer (2004) assuming a cellular volume of 1.4fl, as per Bray (2015)

### Non-dimensionalisation

We re-scale each of the key signalling protein concentrations with respect to their total concentration in the cell, i.e. $[A_p]=a_p[A_T]$, $[B_p]=b_p[B_T]$, and $[Y_p]=y_p[Y_T]$ and time with respect to the rate of spontaneous dephosphorylation of protein CheB-P, i.e. $t=\tau /k_5$. Substitution of these re-scaled variables into Eqs. (5)–(8) yields the system of non-dimensional ODEs

11 $$\begin{aligned} \frac{\hbox {d}m}{d\tau }= & {} \gamma _R(1-\varPhi )-\gamma _Bb_p^2\varPhi = f_1(m,b_p), \end{aligned}$$

12 $$\begin{aligned} \frac{\hbox {d}a_p}{d\tau }= & {} \varPhi \bar{k}_1(1-a_p)-\bar{k}_2(1-y_p)a_p-\bar{k}_3(1-b_p)a_p= f_2(m,a_p,y_p,b_p), \end{aligned}$$

13 $$\begin{aligned} \frac{\hbox {d}y_p}{d\tau }= & {} \alpha _1\bar{k}_2(1-y_p)a_p-(\bar{k}_4+\bar{k}_6)y_p = f_3(a_p,y_p), \end{aligned}$$

14 $$\begin{aligned} \frac{\hbox {d}b_p}{d\tau }= & {} \alpha _2\bar{k}_3(1-b_p)a_p-\bar{k}_5b_p = f_4(a_p,b_p), \end{aligned}$$

with the non-dimensional parameters as defined in Table 2.

**Table 2:** Non-dimensional parameter definitions and their values as calculated using Table 1

Symbol	Value
$\bar{k}_1=k_1/k_5$	48.571
$\bar{k}_2=k_2[Y_T]/k_5$	1385.714
$\bar{k}_3=k_3[B_T]/k_5$	6
$\bar{k}_4=k_4[Z_T]/k_5$	8.686
$\bar{k}_5=k_5/k_5$	1
$\bar{k}_6=k_6/k_5$	0.121
$\alpha _1=[A_T]/[Y_T]$	0.814
$\alpha _2=[A_T]/[B_T]$	28.214
$\gamma _R=g_R[R_T]/k_5$	$8.57\times 10^{-3}$
$\gamma _B=g_B[B_T]^2/k_5$	0.352

### Numerical Solution Method

Numerical solutions to Eqs. (11)–(14) using the parameter values detailed in Table 2 were obtained using the inbuilt MATLAB (2017) ODE solver ode15s. This solver was found to be appropriate given the stiffness coefficient of the system for the parameter values detailed in Table 2 was found to be $\lambda _S=4233.31$.

## Steady-State Stability Analysis

### Steady States

Many biological systems have been shown to exhibit multiple equilibrium states (Eissing et al. 2004; Kim et al. 2007). In order to assess whether this is possible in the *E. coli* chemotaxis signalling pathway, we begin by determining the steady states of Eqs. (11)–(14). This leads to

15 $$\begin{aligned} y_p^*= & {} \frac{\alpha _1\bar{k}_2a_p^*}{\alpha _1\bar{k}_2a_p^*+\bar{k}_4+\bar{k}_6}, \end{aligned}$$

16 $$\begin{aligned} b_p^*= & {} \frac{\alpha _2\bar{k_2}a_p^*}{\alpha _2\bar{k_3}a_p^*+\bar{k_5}} \end{aligned}$$

17 $$\begin{aligned} \varPhi ^*= & {} \frac{1}{1+\frac{\gamma _B}{\gamma _R}b_p^{*2}}, \end{aligned}$$

18 $$\begin{aligned} m^*= & {} 2\left[ 1+\ln \left( \frac{1+[L]/K_a^\mathrm{off}}{1+[L]/K_a^\mathrm{on}}\right) -\frac{1}{N}\ln \left( \frac{\gamma _Bb_p^{*2}}{\gamma _R}\right) \right] . \end{aligned}$$

Each of these equations can be rearranged in terms of ${{a}_{p}^*}$ and substituted into Eq. (12) to yield a fifth-order polynomial of the form

19 $$\begin{aligned} p(a_p^*) = 0 = {C_1}a_p^{*5}+C_2a_p^{*4}+{C_3}a_p^{*3}+{C_4}a_p^{*2}+{C_5}a_p^{*}+C_6, \end{aligned}$$

where $C_{1-6}$ are given by

$$\begin{aligned} C_1= & {} -\,g_R[R_T][A_T]^4k_1k_2k_3^3,\\ C_2= & {} -\,g_B[B_T]^3[A_T]^3k_2k_3^3k_5-3g_R[R_T][A_T]^3k_1k_2k_3^2k_5 \\&-\,g_R[R_T][A_T]^3[Z_T]k_1k_3^3k_4-g_B[B_T]^2[A_T]^3[Y_T]k_2k_3^3k_6 \\&-\,g_R[R_T][A_T]^3[B_T]k_2k_3^3k_5-g_B[B_T]^2[A_T]^3[Y_T][Z_T]k_2k_3^3k_4 \\&-\,g_R[R_T][A_T]^3[Y_T][Z_T]k_2k_3^3k_4+g_R[R_T][A_T]^4k_1k_2k_3^3 \\&-\,g_R[R_T][A_T]^3[Y_T]k_2k_3^3k_6-g_R[R_T][A_T]^3k_1k_3^3k_6, \\ C_3= & {} -\,g_B[B_T]^3[A_T]^2k_3^3k_5k_6+3g_R[R_T][A_T]^3k_1k_2k_3^3k_5 \\&-\,3g_R[R_T][A_T]^2k_1k_3^2k_5k_6-g_R[R_T][A_T]^2[B_T]k_3^3k_5k_6 \\&-\,g_R[R_T][A_T]^2[B_T][Z_T]k_3^3k_4k_5-3g_R[R_T][A_T]^2k_1k_2k_3k_5^2\\&-\,2g_R[R_T][A_T]^2[B_T]k_2k_3^2k_5^2+g_R[R_T][A_T]^3[Z_T]k_1k_3^3k_4\\&-\,3g_R[R_T][A_T]^2[Y_T]k_2k_3^2k_5k_6-3g_R[R_T][A_T]^2[Y_T][Z_T]k_2k_3^2k_4k_5\\&-\,g_B[B_T]^3[A_T]^2[Z_T]k_3^3k_4k_5+g_R[R_T][A_T]^3k_1k_3^3k_6\\&-\,3g_R[R_T][A_T]^2[Z_T]k_1k_3^2k_4k_5-g_B[B_T]^2[A_T]^2[Y_T]k_2k_3^2k_5k_6\\&-\,g_B[B_T][A_T]^2[Y_T][Z_T]k_2k_3^2k_4k_5,\\ C_4= & {} -\,2g_R[R_T][A_T][B_T]k_3^2k_5^2k_6-3g_R[R_T][A_T]k_1k_3k_5^2k_6\\&-\,2g_R[R_T][A_T][B_T][Z_T]k_3^2k_4k_5^2-g_R[R_T][A_T]k_1k_2k_5^3\\&+\,3g_R[R_T][A_T]^2[Z_T]k_1k_3^2k_4k_5-g_R[R_T][A_T][B_T]k_2k_3k_5^3\\&+\,3g_R[R_T][A_T]^2k_1k_3^2k_5k_6-3g_R[R_T][A_T][Y_T]k_2k_3k_5^2k_6\\&-\,3g_R[R_T][A_T][Z_T]k_1k_3k_4k_5^2+3g_R[R_T][A_T]^2k_1k_2k_3k_5^2\\&-\,3g_R[R_T][A_T][Y_T][Z_T]k_2k_3k_4k_5^2,\\ C_5= & {} g_R[R_T][A_T]k_1k_2k_5^3-g_R[R_T][B_T][Z_T]k_3k_4k_5^3-g_R[R_T][B_T]k_3k_5^3k_6\\&+\,3g_R[R_T][A_T]k_1k_3k_5^2k_6-g_R[R_T]k_1k_5^3k_6-g_R[R_T][Y_T][Z_T]k_2k_4k_5^3\\&-\,g_R[R_T][Z_T]k_1k_4k_5^3+3g_R[R_T][A_T][Z_T]k_1k_3k_4k_5^2-g_R[R_T][Y_T]k_2k_5^3k_6,\\ C_6= & {} g_R[R_T][Z_T]k_1k_4k_5^3+g_R[R_T]k_1k_5^3k_6, \end{aligned}$$

where overbars have been dropped in what follows for notational convenience. Since there is no generally applicable analytical solution form for quintic equations such as this, we substitute the parameter values from Table 1 into these polynomial coefficients to determine their respective values. Then, by Descartes’ rule of signs, we find that there may only be one positive root and the remaining four will either be negative or in complex conjugate pairs (i.e. not biologically feasible). It is worth noting here that the steady-state expressions for each key signalling protein are independent of the average chemoreceptor methylation level (*m*) and the extracellular chemoattractant concentration ([*L*]). This differs from the expression for $m^*$ which depends upon the extracellular chemoattracant concentration as well as the CheB-P steady state ($b_p^*$).

**Fig. 2:**
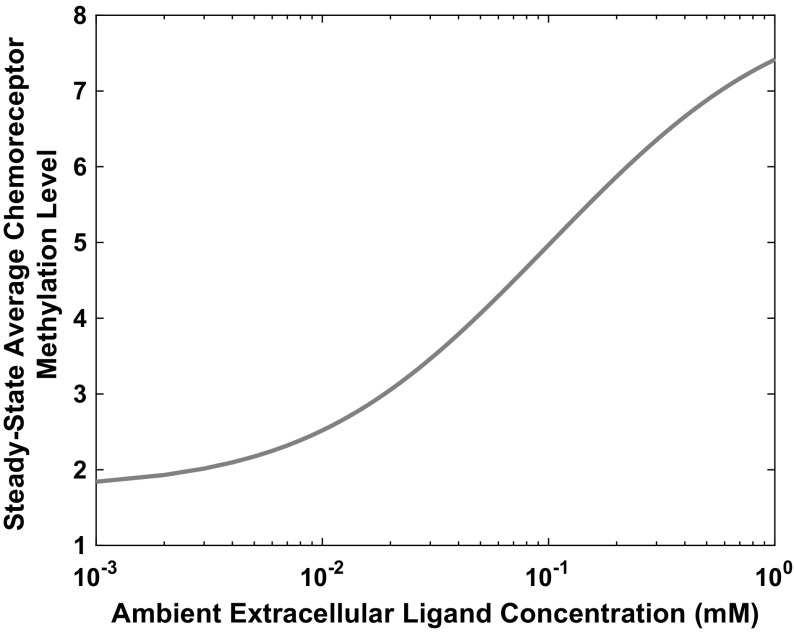
(Color figure online) Plot showing how the steady-state value for the average chemoreceptor methylation level rises in relation to the ambient extracellular ligand concentration. This result is similar to those given by Hansen et al. (2008) and Endres and Wingreen (2006)

Using the steady-state expressions (15)–(19) and the parameter values listed in Tables 1 and 2, we obtain the following non-dimensional steady-state values

$$\begin{aligned} a_p^*=5.58{\times }10^{-3},\quad b_p^*=4.86{\times }10^{-1},\quad y_p^*=4.17{\times }10^{-1}, \end{aligned}$$

given to three significant figures. The steady-state methylation level is dependent upon the extracellular chemoattractant concentration and rises or falls in order to reset the protein phosphorylation levels ($a_p$, $y_p$ and $b_p$) to their pre-stimulus values. This is defined by Eq. (18) and demonstrated in Fig. 2.

### Stability Analysis

We now determine the asymptotic stability of the steady state determined in Sect. 3.1. The Jacobian matrix for the system of Eqs. (11)–(14) is given by

20 $$\begin{aligned} J=\left( \begin{array}{l@{\quad }l@{\quad }l@{\quad }l} \frac{\partial f_1}{\partial m} &{} 0 &{} 0 &{} \frac{\partial f_1}{\partial b_p} \\ \frac{\partial f_2}{\partial m} &{} \frac{\partial f_2}{\partial a_p} &{} \frac{\partial f_2}{\partial y_p} &{} \frac{\partial f_2}{\partial b_p} \\ 0 &{} \frac{\partial f_3}{\partial a_p} &{} \frac{\partial f_3}{\partial y_p} &{} 0 \\ 0 &{} \frac{\partial f_4}{\partial a_p} &{} 0 &{} \frac{\partial f_4}{\partial b_p} \end{array}\right) , \end{aligned}$$

where

$$\begin{aligned} \frac{\partial f_1}{\partial m}= & {} \frac{-Ne^F\varPhi ^2}{2k_5}\left( g_R[R_T]+g_B[B_T]^2b_p^2\right) ,\\ \frac{\partial f_1}{\partial b_p}= & {} \frac{-2g_B[B_T]^2b_p\varPhi }{k_5},\\ \frac{\partial f_2}{\partial m}= & {} \frac{Nk_1(1-a_p)e^F\varPhi }{2k_5},\\ \frac{\partial f_2}{\partial a_p}= & {} \frac{k_1\varPhi }{k_5}-\frac{k_2[Y_T](1-y_p)}{k_5}-\frac{k_3[B_T](1-b_p)}{k_5},\\ \frac{\partial f_2}{\partial y_p}= & {} \frac{k_2[Y_T]a_p}{k_5},\\ \frac{\partial f_2}{\partial b_p}= & {} \frac{k_3[B_T]a_p}{k_5},\\ \frac{\partial f_3}{\partial a_p}= & {} \frac{k_2[A_T](1-y_p)}{k5},\\ \frac{\partial f_3}{\partial y_p}= & {} \frac{-k_2[A_T]a_p}{k_5}-\frac{k_4[Z_T]}{k_5}-\frac{k_6}{k_5},\\ \frac{\partial f_4}{\partial a_p}= & {} \frac{k_3[A_T](1-b_p)}{k_5},\\ \frac{\partial f_4}{\partial b_p}= & {} \frac{-k_3[A_T]a_p}{k_5}-1. \end{aligned}$$

The eigenvalues of the system are determined by solving

21 $$\begin{aligned} \det |J-\lambda I|=0, \end{aligned}$$

in which *I* is the identity matrix with equal dimensions to the Jacobian matrix and $\lambda$ denotes an eigenvalue. Solving this equation leads to a quartic polynomial in $\lambda$ from which it is difficult to identify the nature of the eigenvalues analytically given the large and complex form of the respective coefficients (details not shown). As such, we again utilise the parameter values given in Table 1 which leads to the four eigenvalues

$$\begin{aligned} \lambda _1=-822.1086, ~~ \lambda _2=-8.8146, ~~ \lambda _3=-1.7996, ~~ \hbox {and} ~~ \lambda _4=-0.1942. \end{aligned}$$

These are each real and negative, and hence we conclude that this steady state is asymptotically stable for the parameter values given in Table 1.

## Oscillations and the CheB-P Negative Feedback

It is clear that the base parameter set given in Table 1 produces an asymptotically stable equilibrium state. However, it is interesting to note the existence of a negative feedback loop in which CheB-P acts to reduce the methylation level of the cell’s chemoreceptors. Such negative feedbacks have been shown to have potential for creating oscillatory behaviour within monotone systems (Pigolotti et al. 2007; Snoussi 1998; Gouze 1998). Furthermore, variation of kinetic rate constants is known to be limited, whilst total cell protein levels are known to vary significantly between cells (Elowitz et al. 2002; Li and Hazelbauer 2004). We now consider whether variation in the respective parameters and total cell concentrations could possibly induce such behaviour within the pathway.

In order to test this hypothesis, we examined pair-wise variations in both kinetic rate parameters and total signalling protein concentrations. Specifically, we varied each pair of kinetic rate parameters and total protein concentrations over a tenfold range in respect of the parameter values given in Table 1. The stability analysis of Sect. 3.2 was repeated for variations in the parameters, whereby for each case the occurrence of at least two non zero imaginary parts was recorded as indicating oscillatory dynamics. Here, we limited our consideration to pair-wise parameter variations in order to aid both visualisation and interpretation of results.

**Fig. 3:**
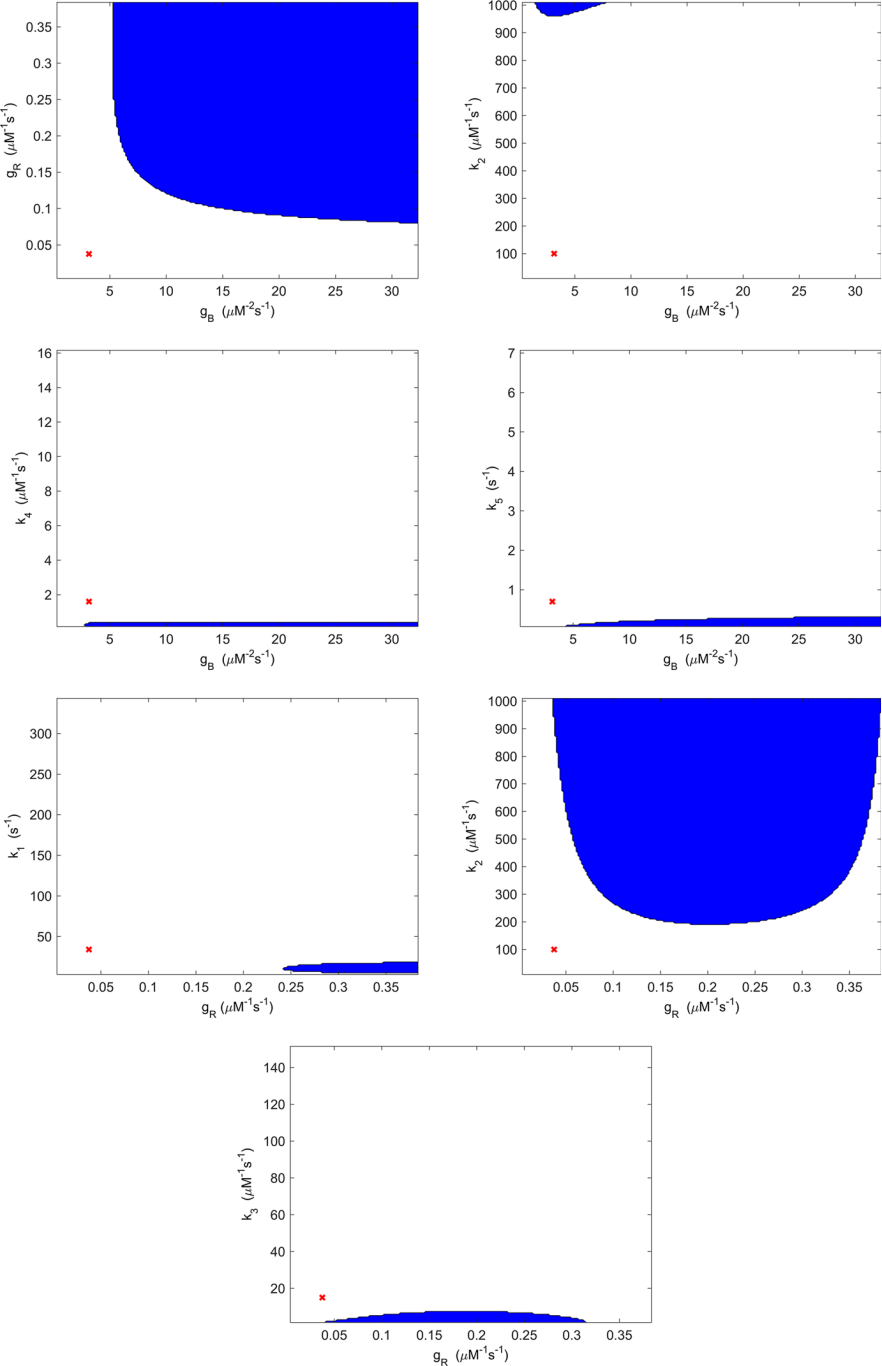
(Color figure online) Regions of parameter space in which oscillatory behaviour may be found by varying kinetic rate parameters (blue). Regions indicated are those in which at least two eigenvalues of the system have nonzero imaginary part. Red crosses indicate the location of the parameters detailed in Table 1

**Fig. 4:**
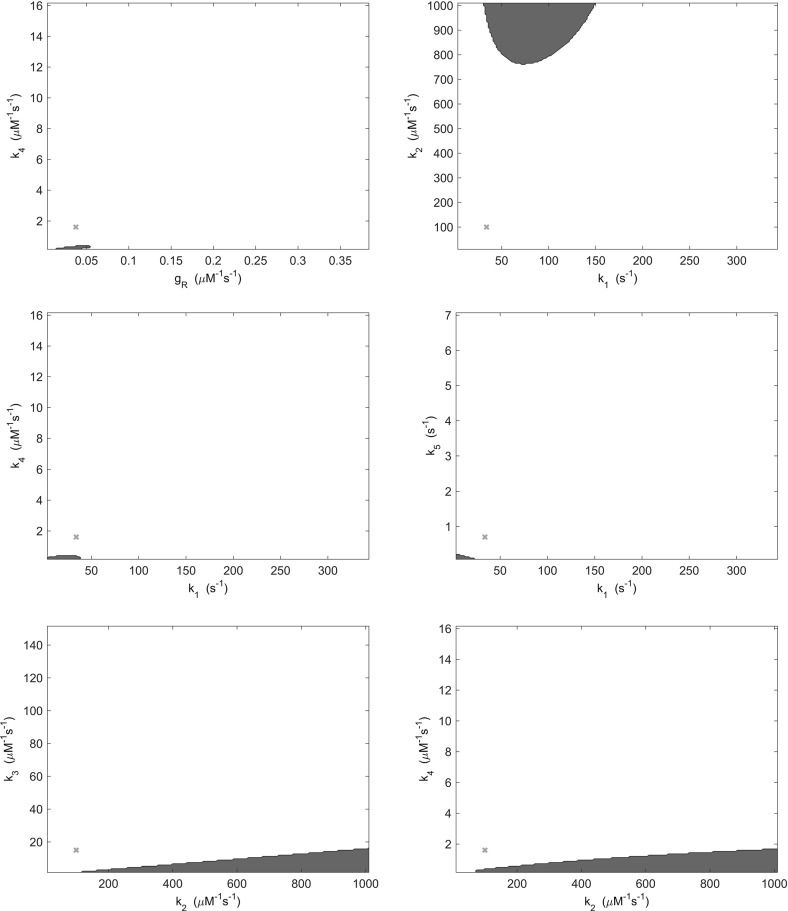
(Color figure online) Regions of parameter space in which oscillatory behaviour may be found by varying kinetic rate parameters (blue). Regions indicated are those in which at least two eigenvalues of the system have nonzero imaginary part. Red crosses indicate the location of the parameters detailed in Table 1

**Fig. 5:**
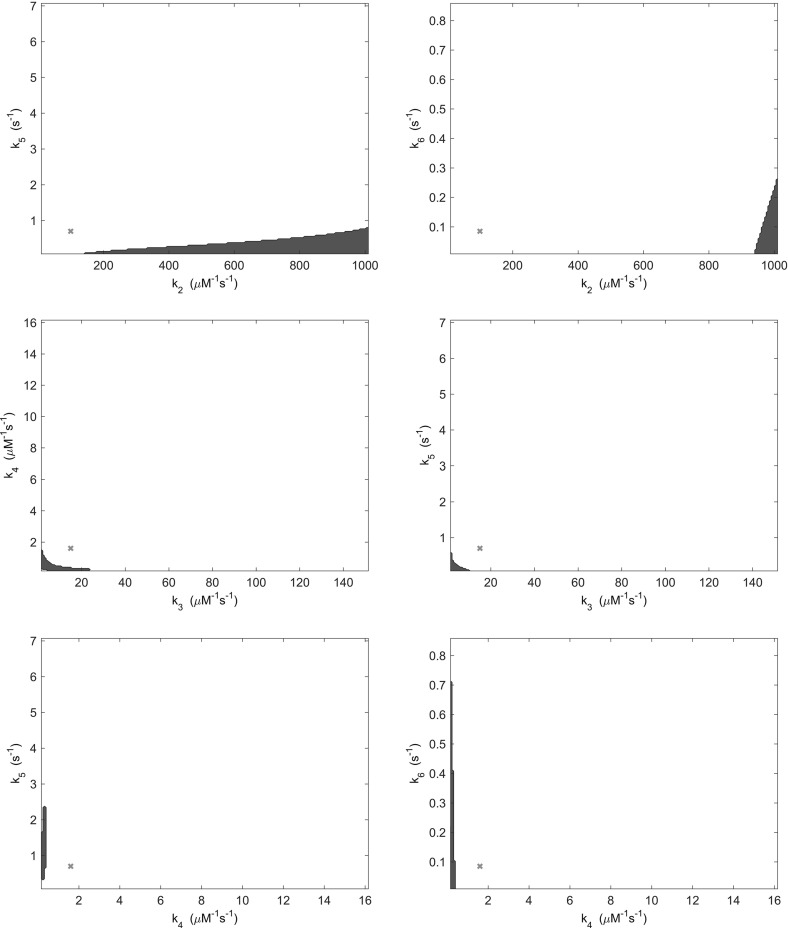
(Color figure online) Regions of parameter space in which oscillatory behaviour may be found by varying kinetic rate parameters (blue). Regions indicated are those in which at least two eigenvalues of the system have nonzero imaginary part. Red crosses indicate the location of the parameters detailed in Table 1

Results obtained when varying pairs of kinetic rate parameters demonstrate that 9 of 28 possible pairs produced no oscillatory behaviour within a tenfold range. The remaining 19 pairs, as shown in Figs. 3, 4 and 5, do yield some oscillatory behaviour, but in regions of parameter space outside that observed experimentally. This is due to the fact that each protein molecule would be expected to carry out each process in which it is involved at an equal rate. Thus, kinetic rates are unlikely to vary far enough from the base parameter set of Table 1 in order to yield oscillatory behaviour. It therefore seems unlikely that variation in kinetic rate parameters produces experimentally observable oscillatory behaviour in *E. coli* cells.

**Fig. 6:**
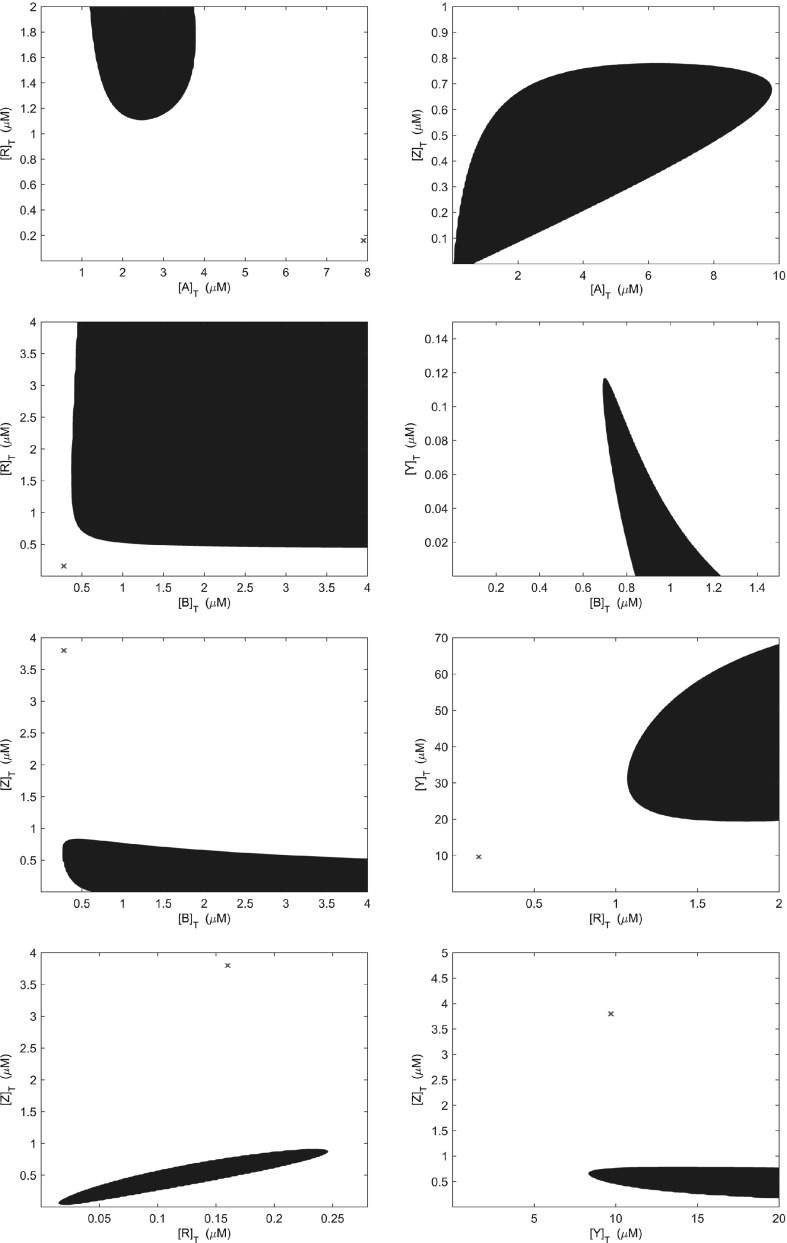
(Color figure online) Regions of parameter space in which oscillatory behaviour may be found by varying the total concentration of each chemotaxis proteins. Regions shown are those in which at least two eigenvalues of the system have a nonzero imaginary part. The colours of the contour lines represent the magnitudes of the imaginary parts of the eigenvalues obtained from the fourth-order system. *Note*: where a red cross appears this indicates the location of our base parameter set. All concentration axes are expressed in $\upmu \hbox {M}$

Repeating this same methodology for pair-wise variations in total protein concentrations reveals that 8 of 10 pairs are able to produce oscillatory behaviour within the range tested, as shown in Fig. 6. Interestingly, it may be observed that pair-wise variations in each chemotaxis signalling protein showed that CheB, CheR, CheY and CheZ allow oscillatory behaviour to occur more readily than combinations involving CheA. Of particular interest here is the case in which CheB and CheY are varied together. In this case, total concentrations of CheB and CheY must be increased and decreased from the experimentally measured population average values in Table 1. This suggests that the ratio of CheB and CheY concentrations may be important for producing oscillatory solutions. Since phosphoryl groups are passed from CheA-P to both CheB and CheY, the ratio of these total protein concentrations will clearly affect how many phosphoryl groups are passed to each protein. As such, we postulate here that this will affect both the timescale and strength of CheB-P feedback on the receptor state, thus leading to the emergence of oscillatory behaviour.

**Fig. 7:**
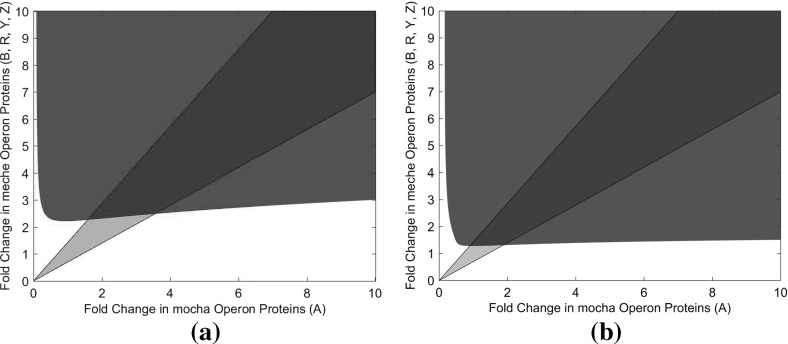
(Color figure online) Operon-wise variation in total protein concentrations within the signalling cascade model can produce oscillatory behaviour. Plots showing the regions in which oscillatory behaviour may be obtained when considering methylation/demethylation kinetics defined by (**a**) Eq. (8) and (**b**) Eq. (22). The biologically feasible region is shaded in grey

Experimentally, it is known that proteins of the *E. coli* chemotaxis signalling pathway are encoded in two operons, namely *mocha* (CheA and CheW) and *meche* (CheB, CheR, CheY and CheZ) (Kalir 2001). This is a key mechanism by which cells are able to maintain suitable protein levels and ratios. In these groupings, we would expect the ratios of proteins encoded in the same operon to maintain approximately fixed ratios, whilst more variation is expected to exist between proteins encoded in different operons. We therefore group proteins by operon and allow variations over a tenfold range. This entails varying the total concentration of CheA against concentrations of CheB, CheR, CheY and CheZ which were varied so as to maintain a constant ratio within each group, the result of which is shown in Fig. 7. We found here that an increase of $\sim$threefold in all protein concentrations was sufficient to yield oscillatory behaviour, falling well within the biologically realistic tenfold variation.

Whilst individual total protein concentrations have been found to vary by as much as tenfold, ratios between them have been shown to vary by approximately 30% (Li and Hazelbauer 2004). We would expect the maximal variation to occur between proteins encoded by separate operons since protein co-expression will limit stochastic fluctuation in ratios of proteins encoded within the same operon. Thus, a biologically feasible range within Fig. 7 would be 0.7$\alpha \le \beta \le 1.3\alpha$ in which $\alpha$ represents the fold change in the proteins of one operon and $\beta$ the fold change in proteins of the remaining operon. We note that beyond an increase of $\sim$fourfold in the concentration of CheA, the vast majority of change in the magnitude of the imaginary parts of the eigenvalues appears to be caused by variation in proteins encoded by the *meche* operon. This is supported by the earlier finding that pairs of proteins involving CheB, CheR, CheY and CheZ are more readily able to produce oscillatory behaviour than those including CheA.

A major assumption of the model considered here is that the rates of chemoreceptor methylation/demethylation are linear functions of the receptor-kinase activity (Eq. 8). This differs from a number of previous models in which these rates are described by Michaelis–Menten kinetics (Barkai and Leibler 1997; Clausznitzer et al. 2010; Emonet and Cluzel 2008; Tu et al. 2008). Such models have been shown to fit both population (Shimizu et al. 2010) and single cell (Emonet and Cluzel 2008) measurements. As such, we also investigated the occurrence of oscillations when receptor methylation/demethylation is described by Michaelis–Menten kinetics such that

22 $$\begin{aligned} \frac{\hbox {d}m}{\hbox {d}t} = g_R\frac{1-\varPhi }{1-\varPhi +K_1} - g_B[B_p]^2\frac{\varPhi }{\varPhi +K_2}, \end{aligned}$$

with $K_1=0.0229$ and $K_2=0.0318$ as per the Supplementary Information of Clausznitzer et al. (2010). In Fig. 7b, we found similar results to those shown in Fig. 7a with the exception that a smaller fold increase ($\sim$ 1.5-fold) was required to produce oscillatory behaviour.

## Oscillatory Dynamics and the Overshoot Phenomenon

The possible occurrence of oscillations in the temporal expression of signalling proteins within the signalling network raises the question as to whether this phenomenon may be observed experimentally. Overshoot, as demonstrated by the dotted line [CheY-P] response in Fig. 1, has been observed both experimentally and theoretically within certain *E. coli* studies. In the context of our work, an overshoot response is equivalent to underdamped oscillations.

Within the literature, the phenomenon of overshoot has been linked to the likely interaction between different chemoreceptor types. In particular, past theoretical work has proposed overshoot to be caused by crosstalk in receptor methylation levels (Hansen et al. 2010; Lan et al. 2011). Our results suggest that whilst methylation and demethylation play an important role, it is the coupling of this process with the negative CheB-P feedback that is the overall driver of the observed overshoot response. This in turn is a result of protein concentrations within the signalling network. It could also be possible that crosstalk in receptor methylation acts in tandem with the mechanism proposed here in order to produce experimentally observed overshoot amplitudes. We propose that methylation crosstalk could set a basal level of overshoot, whilst the mechanism proposed here acts to amplify this in order to achieve the larger overshoot amplitudes observed for intermediate ligand stimuli.

**Fig. 8:**
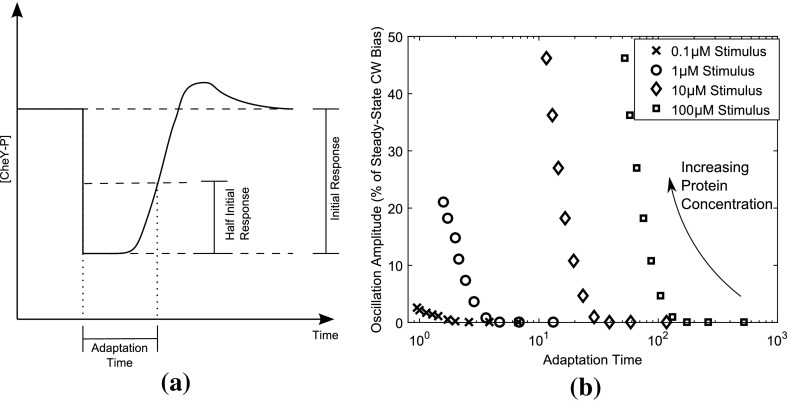
Plot showing the relationship between adaptation time, the magnitude of the ligand stimulus and intracellular protein concentration. (**a**) Here, the adaptation time is chosen to be the time necessary for a cell to recover from half of the initial response, determined from numerical simulations of the full fourth-order model. (**b**) Shown here are the overshoot amplitudes and associated adaptation times for different size of step-up ligand stimuli, namely 0.1 $\upmu \hbox {M}$ (represented by crosses), 1 $\upmu \hbox {M}$ (circles), 10 $\upmu \hbox {M}$ (diamonds) and 100 $\upmu \hbox {M}$ (squares). The different data points for each stimulus refer to simulated cells with different (1–10) fold increases in the total concentration of all proteins. Shorter adaptation times are associated with larger fold increases in all total protein concentrations. Clearly, cells with shorter adaptation times display larger overshoot amplitudes; however, there is also a dependence on the size of ligand stimulus applied, as noted by Min et al. (2012)

Coupled with the role of receptor methylation and the CheB-P negative feedback is the overall ligand concentrations a cell may experience. Recent experimental work by Min et al. (2012) has focused on the overshoot response in the context of varying ligand concentrations. This investigation and the relationship here between the total concentration of proteins within the network and the occurrence of oscillatory behaviour raise the question as to how these may be linked. As such, we have considered the effect of varying ligand concentrations whilst perturbing the overall concentration of the signalling proteins as shown in Fig. 8. These results demonstrate that the overshoot amplitude increases with total protein concentration and this relationship holds for a range of ligand concentrations. However, whilst the overshoot amplitude increases, the adaptation time subsequently decreases as an increase in phosphotransfer from CheA-P onto CheB (forming CheB-P) allows the negative feedback to act more quickly.

## Model Reduction Analysis

**Fig. 9:**
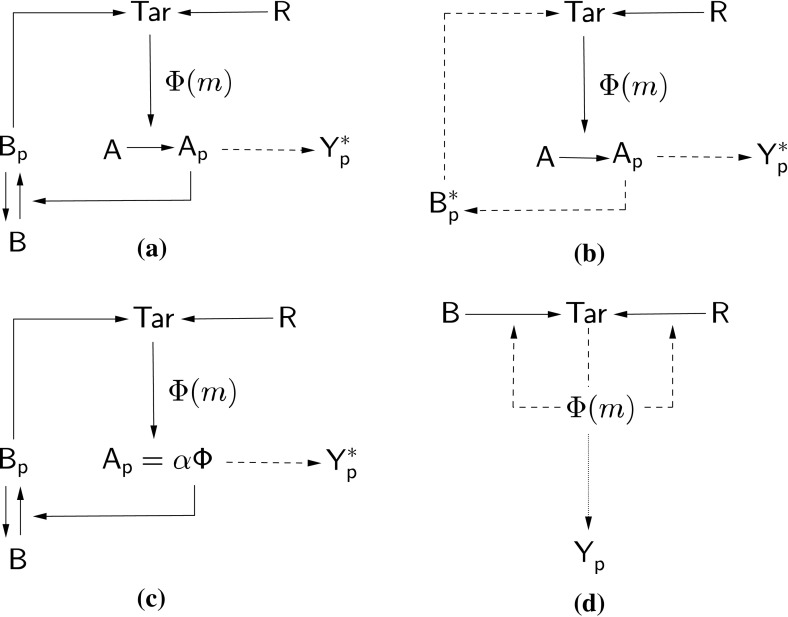
Schematic representations of the four model reductions considered. (**a**) Reduction to a third-order system by applying the quasi-steady-state approximation to CheY-P. (**b**) Reduction to a second-order system via application of the quasi-steady-state approximation to both CheY-P and CheB-P. (**c**) Reduction to a second-order system by assuming CheA-P may be represented by a multiple scaling of receptor signalling team activity (i.e. $A_p=\alpha \varPhi$) and representing CheY-P as a decouplable read-out variable. (**d**) A first-order model due to Tu et al. (2008). Here, solid lines indicate interactions, whilst dashed lines indicate quasi-steady-state/read-out variables. The dotted line in (**d**) represents the decoupled expression for CheY-P

In light of the results of Sect. 4, we wish to further our understanding of how the CheB-P feedback behaves in the context of variation in total protein concentration and how different timescales within the signalling cascade affect the overshoot response. We are also motivated to understand why some nonlinear ODE mathematical models describing *E. coli* chemotaxis exhibit overshoot, whilst others do not and how their formulations and dimensionality may affect this. As such, we consider here a variety of further reductions to the fourth-order model system (11)–(14). These are illustrated in Fig. 9. In what follows, we begin by considering the third-order reduction in Eqs. (11)–(14) in Sect. 6.1, moving to a range of second-order cases in Sect. 6.2. Doing so allows us to compare the various reductions with similar models in the literature, identify key features of the system which drive the overall signalling output and may or may not be responsible for overshoot like responses.

### Third-Order System

Motivated by the non-dimensional parameter values in Table 2, we assume here that CheY-P reaches a steady state rapidly, in comparison with all other proteins, such that the quasi-steady-state approximation (QSSA) holds. This leads to the third-order dynamical system

23 $$\begin{aligned} \frac{\hbox {d}m}{d\tau }= & {} \frac{g_R[R_T]}{k_5}(1-\varPhi )-\frac{g_B[B_T]^2}{k_5}b_p^2\varPhi , \end{aligned}$$

24 $$\begin{aligned} \frac{\hbox {d}a_p}{d\tau }= & {} \varPhi \frac{k_1}{k_5}(1-a_p)-\frac{k_2[Y_T]}{k_5}(1-y_p^*)a_p - \frac{k_3[B_T]}{k_5}(1-b_p)a_p, \end{aligned}$$

25 $$\begin{aligned} \frac{\hbox {d}b_p}{d\tau }= & {} \frac{k_3[A_T]}{k_5}(1-b_p)a_p-b_p, \end{aligned}$$

within which $y_p^*$ denotes the non-dimensional steady state for CheY-P, as given by Eq. (15). The corresponding network is shown in Fig. 9a.

**Fig. 10:**
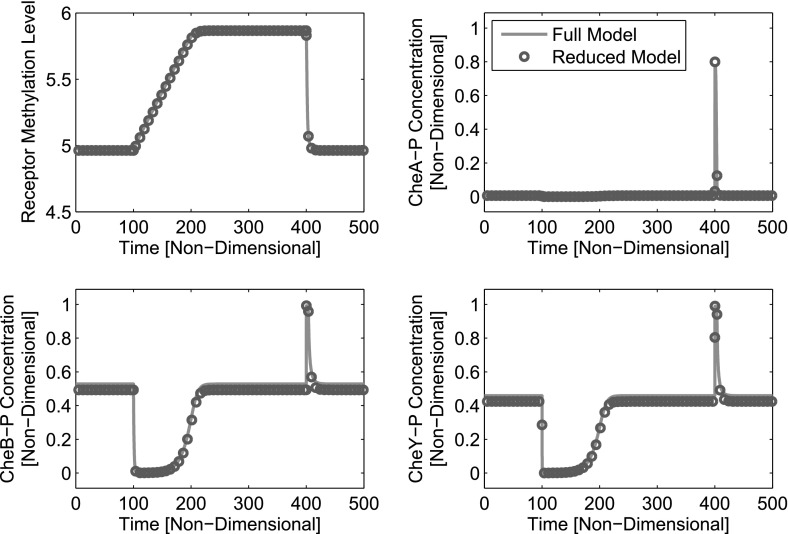
(Color figure online) Comparison of the full fourth-order (blue lines) and the reduced third-order (red circles) systems

The application of this reduction only slightly changes the steady-state values of the system (Fig. 10). As such, we now test the stability characteristics of this system by analysing the eigenvalues of its Jacobian matrix

26 $$\begin{aligned} J=\left( \begin{array}{l@{\quad }l@{\quad }l} f_m &{} 0 &{} f_{b_p} \\ g_m &{} g_{a_p} &{} g_{b_p} \\ 0 &{} h_{a_p} &{} h_{b_p} \end{array} \right) , \end{aligned}$$

within which

$$\begin{aligned} f_m= & {} \frac{-g_R[R_T]Ne^F\varPhi ^2-g_B[B_T]^2b_p^2Ne^F\varPhi ^2}{2k_5},\\ f_{b_p}= & {} \frac{-2g_B[B_T]^2b_p\varPhi }{k_5},\\ g_m= & {} \frac{k_1(1-a_p)Ne^F\varPhi ^2}{2k_5},\\ g_{a_p}= & {} \frac{-1}{k_5}\left( k_1\varPhi +k_2[Y_T](1-y_p^*)+k_2[Y_T]\left( y_p^{*2}-y_p^*\right) +k_3[B_T](1-b_p)\right) ,\\ g_{b_p}= & {} \frac{k_3[B_T]a_p}{k_5},\\ h_{a_p}= & {} \frac{k_3[A_T](1-b_p)}{k_5},\\ h_{b_p}= & {} \frac{-k_3[A_T]a_p}{k_5}-1 \end{aligned}$$

are the partial derivatives of Eqs. (23)–(25) with respect to each of the three variables. In order to obtain the eigenvalues of the system, it is necessary to find the characteristic polynomial of this Jacobian matrix. In this case, the characteristic polynomial is given by

27 $$\begin{aligned} p(\lambda ) = \left| \begin{array}{l@{\quad }l@{\quad }l} f_m-\lambda &{} 0 &{} f_{b_p} \\ g_m &{} g_{a_p}-\lambda &{} g_{b_p} \\ 0 &{} h_{a_p} &{} h_{b_p}-\lambda \end{array} \right| . \end{aligned}$$

We now form the characteristic polynomial $p(\lambda )=\lambda ^3+A\lambda ^2+B\lambda +C$ in which *A*,  *B* and *C* are polynomial coefficients and $\lambda$ is an eigenvalue of the system. Here, we define $A=3a$, $B=3b$, $\alpha =a^2-b$ and $\beta =2a^3-3ab+C$ (Murray 2002). The relative sizes of $A, B, \alpha$ and $\beta$ determine the solution form appropriate for the polynomial $p(\lambda )$. Since we are looking to explain the emergence of oscillatory behaviour, we restrict our attention to combinations of $A, B, \alpha$ and $\beta$ which yield a pair of eigenvalues with negative real and nonzero imaginary parts. Using the parameter values in Table 1, we find that $\beta >2\alpha ^{3/2}$ must hold in order for us to obtain oscillatory behaviour. We then apply the condition $\beta >2\alpha ^{3/2}$ to our third-order model and use the parameter values (Table 1) to examine the magnitudes of each term in the expression. Upon doing so, we find that all terms lie in the range $O(10^4)$ to $O(10^7)$ and retaining all terms $>O(10^4)$ leads to the expression

28 $$\begin{aligned} \hbox {Re}\left( \frac{2}{27}k_2^3[Y_T]^3(1-y_p^*)^3\left[ 1-\left( 1-x\right) ^{3/2}\right] \right) >5\times 10^5, \end{aligned}$$

in which

29 $$\begin{aligned} x=\frac{\frac{3N}{2}e^F\varPhi ^2\left( g_R[R_T]+g_B[B_T]b_p^{*2}\right) +k_3[A_T]a_p^*+k_5}{k_2[Y_T](1-y_p^*)}. \end{aligned}$$

Here, $5\times 10^5$ is an approximation of the magnitude of the largest term less than $O(10^7)$. Within this expression, *N* is the number of receptors in a signalling team which has activity $\varPhi$, *F* represents the free energy of a Tar receptor, $[{\ldots }_T]$ denotes the total concentration of the relevant protein, and $a_p^*$ and $b_p^*$ are the (non-dimensional) steady-state concentrations of proteins CheA-P and CheB-P, respectively.

**Fig. 11:**
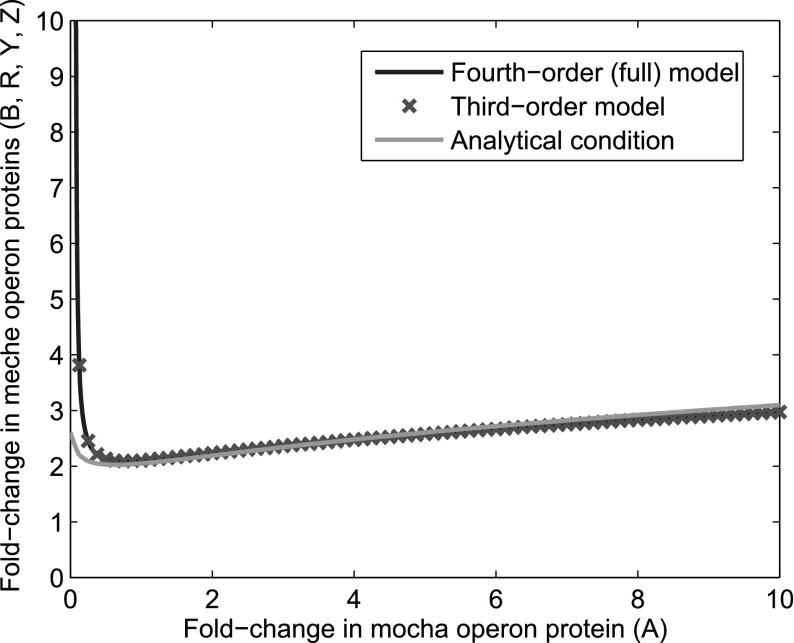
(Color figure online) Comparison of numerical and analytical approximations to the region in which oscillatory behaviour is found. The area above each of these lines signifies the region in which the relevant model exhibits oscillatory behaviour. The blue line indicates the region of oscillatory behaviour found from the full fourth-order dynamical system. Red crosses show the region in which oscillatory behaviour is found in the third-order case in which the quasi-steady-state approximation has been applied to the concentration of CheY-P. Finally, the green line shows the region predicted by the analytical condition given by Eq. (28)

Equation (28) compares well to numerical simulations of the fourth- and third-order systems as shown in Fig. 11, suggesting that the expression captures the key processes involved in causing oscillatory behaviour. Indeed, it includes terms associated with receptor dynamics, the feedback timescale of CheB-P and phosphotransfer to CheY, suggesting that these each play a role in the emergence of oscillatory behaviour.

In an attempt to narrow down the causes of oscillatory behaviour, we separately examine the numerator of Eq. (28). We begin by neglecting $(2/27)k_2^3[Y_T]^3(1-y_p^*)^3$ since it is an approximately exponential multiplier, when subjected to simultaneous, equal fold changes to the concentration of all total protein concentrations. To leading order, an asymptotic expansion of the remaining terms (assuming $x\ll 1$) gives

$$\begin{aligned} 1-(1-x)^{3/2}\approx \frac{3}{2}x+{\cdots }, \end{aligned}$$

where ${\ldots }$ indicates the addition of lower-order terms. This leaves 3*x* / 2 [where *x* is given by Eq. (29)]. This can be further simplified upon neglecting ${(2/3)k_2[Y_T](1-y_p^*)}$ as it is approximately linear for equal fold changes in all total protein concentrations. We therefore restrict our attention to

30 $$\begin{aligned} \psi =\underbrace{\frac{3N}{2}e^F\varPhi ^2\left( g_R[R_T]+g_B[B_T]^2b_p^{*2}\right) }_{\hbox {Receptor dynamics}}+\underbrace{k_3[A_T]a_p^*+k_5}_{\hbox {CheB-P feedback}}. \end{aligned}$$

**Fig. 12:**
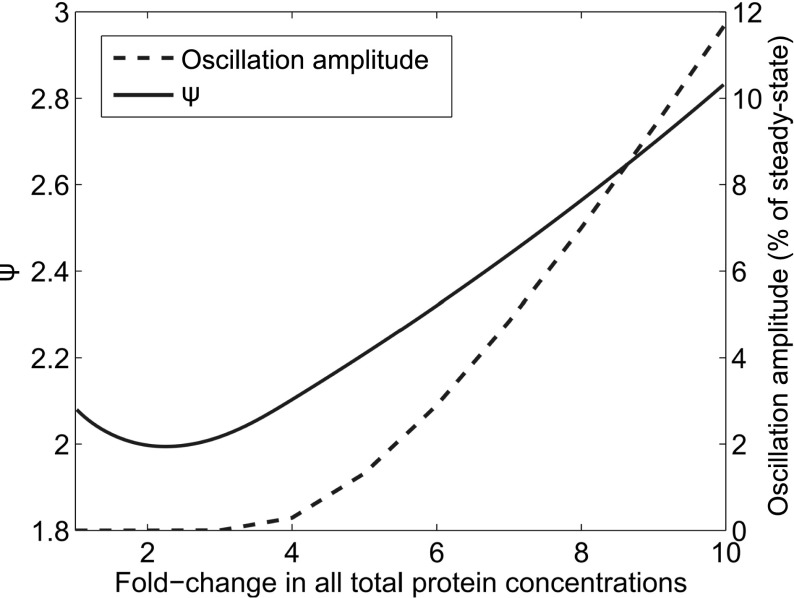
(Color figure online) Receptor dynamics and CheB-P feedback timescale are critical in the emergence of oscillatory behaviour within the mathematical model. The solid line shows values obtained from Eq. (30), and dashed lines show amplitudes of the first oscillation calculated from numerical simulations. These amplitudes are obtained under equal fold changes in the total concentrations of all chemotaxis signalling proteins and are expressed as a percentage of the steady-state CheY-P concentration. The location of the minimum of the solid line corresponds to the fold change required in order to obtain a nonzero oscillation amplitude

Figure 12 shows results obtained from $\psi$ in addition to amplitudes of the first oscillations calculated from numerical simulations of the third-order system and obtained under the same equal fold changes in all total protein concentrations. Interestingly, we observe that the minimum of the curve given by Eq. (30) corresponds to the initial fold increase in total concentration of all chemotaxis signalling proteins at which a non zero oscillation amplitude is observed. We note here that the first underbrace of Eq. (30) shows that the receptor state, specifically that the methylation and demethylation rates are important. Also highlighted, in the second underbrace of Eq. (30), was the importance of CheB-P feedback onto the receptor state, as shown by the presence of $k_3$, $k_5$, $[A_T]$ and $a_p^*$. Specifically, $[A_T]a_p^*$ represents the number of phosphoryl groups available for transfer from CheA-P onto CheB at steady state, whilst $k_3$ shows how quickly phosphoryl groups may be transferred around the system, causing demethylation of receptors. This leads us to conclude that the balance between receptor dynamics and CheB-P feedback is key in determining whether or not an oscillatory response is observed.

### Second-Order Systems

#### Rapid Equilibrium of CheB-P and CheY-P

Based on the validity of the third-order model reduction, we seek here to extend the application of the QSSA. Taking the third-order system and examining the remaining parameter values, we find that the rate of phosphotransfer from CheA-P to CheB is quite large in comparison with most other values. As such, we consider the application of the QSSA to the protein CheB-P (Fig. 9b), thereby producing a second-order system of the form

31 $$\begin{aligned} \frac{\hbox {d}m}{d\tau }= & {} \frac{g_R[R_T]}{k_5}(1-\varPhi )-\frac{g_B[B_T]^2}{k_5}b_p^{*2}\varPhi ,\end{aligned}$$

32 $$\begin{aligned} \frac{\hbox {d}a_p}{d\tau }= & {} \varPhi \frac{k_1}{k_5}(1-a_p)-\frac{k_2[Y_T]}{k_5}(1-y_p^*)a_p - \frac{k_3[B_T]}{k_5}(1-b_p^*)a_p, \end{aligned}$$

where $y_p^*$ and $b_p^*$ are given by Eqs. (15) and (16), respectively.

Analysing the stability of this system, we found that the region of parameter space (total protein concentrations only) in which oscillations are found was altered significantly. Specifically, much larger total protein concentrations were required for this reduced model to exhibit oscillatory behaviour. This is likely due to the removal of features related to the negative feedback of CheB-P on the chemoreceptors, suggesting that it is important for oscillatory behaviour to be observed.

#### CheA-P as a Scaling of Receptor Signalling Team Activity

An alternate second-order reduction assumes that CheY-P is a decoupled output variable as detailed in Tu et al. (2008). We also consider the concentration of CheA-P in the system to be a simple multiplicative scaling of the receptor signalling team activity, i.e. $\sigma \varPhi \approx [A_p]$ (Fig. 9c). Here, $\sigma$ is calculated at steady state from a numerical simulation of the full system using parameters from Table 1. These assumptions produce a second-order model of the form

33 $$\begin{aligned} \frac{\hbox {d}m}{d\tau }= & {} \frac{g_R[R_T]}{k_5}(1-\varPhi )-\frac{g_B[B_T]^2}{k_5}b_p^2\varPhi ,\end{aligned}$$

34 $$\begin{aligned} \frac{\hbox {d}b_p}{d\tau }= & {} \frac{k_3}{k_5}\sigma \varPhi (1-b_p)-b_p. \end{aligned}$$

In terms of the network structure, this reduced model had two main implications. Firstly, the decoupling of CheY-P means there is no longer competition for phosphoryl groups from CheA-P. This means CheY receives phosphoryl groups regardless of the competitive ability of CheB. Furthermore, the timescale of CheA autophosphorylation was eliminated by considering CheA-P to be a scaling of receptor signalling team activity.

Analysis showed that a large fold change ($\sim$ 9.5-fold increase) in all total protein concentrations was required for this model to display any oscillatory behaviour. This was supportive of the notion that timescales and sharing of phosphoryl groups around the system may both be important features of the mechanism regulating the emergence of oscillatory behaviour.

#### Tu et al. Model

Finally, we consider the model defined by Tu et al. (2008) (Fig.9d). This is a second-order system reduction with

35 $$\begin{aligned} \frac{\hbox {d}m}{\hbox {d}t} = k_\mathrm{cat}^R[R_T]\frac{1-\varPhi }{1-\varPhi +K_M^R}-k_\mathrm{cat}^B[B_T]\frac{\varPhi }{\varPhi +K_M^B}, \end{aligned}$$

in which $k_\mathrm{cat}^{R/B}$ and $K_M^{R/B}$ are the catalytic rates and Michaelis–Menten constants of CheR and CheB, respectively. Here, the concentration of CheY-P is described by

36 $$\begin{aligned} \frac{\hbox {d}[Y_p]}{\hbox {d}t} = k_a\varPhi - \frac{[Y_p]}{\tau _z}, \end{aligned}$$

in which $k_a$ is the rate of phosphotransfer from CheA-P onto CheY and $\tau _z$ is the dephosphorylation time of the protein CheY-P. This model is based upon a number of assumptions including CheB acting only on active receptors, CheR only acting upon inactive receptors and that CheY-P decouples from Eq. (35).

Mathematically, we need only investigate Eq. (35) since Eq. (36) decouples. Analysis revealed that no oscillatory behaviour is possible within the range tested (tenfold increase and decrease in protein concentrations varied in operon groupings). We note that this model includes only the total concentration of proteins CheR and CheB, the implication being that the phosphorylated fraction of CheB is unimportant in determining the receptor state. However, as this model does not capture the stability characteristics of the fourth-order system we are led to the conclusion that the feedback of CheB-P onto the receptor state is critical in producing oscillations.

## Discussion

In this paper, we have undertaken a detailed numerical and analytical investigation of a nonlinear ODE model of the *E. coli* chemotactic signalling cascade (Clausznitzer et al. 2010). This model includes a description of the signalling pathway biochemistry and an average description of the receptor methylation/demethylation dynamics. Our work has shown that the known network structure, receptor state, dynamic timescales and sharing of phosphoryl groups within the *E. coli* chemotaxis signalling pathway may act in unison to produce oscillatory behaviour. In particular, examining the effects of operon-wise variation in total signalling protein concentrations, we found a balance between receptor state and the timescale of CheB-P receptor demethylation to be a key feature responsible for oscillations to be observed.

The observed phenomenon of overshoot in the *E. coli* response is equivalent to underdamped oscillations within the context of our studies. As such, we have sought to elucidate the relationship between extracellular ligand concentrations, total protein concentration levels within the cell and the overshoot (damped oscillation) amplitude. Our results indicate that the overshoot response is a combination of the CheB-P negative feedback onto the receptor state and the total concentration of proteins within the signalling network. As the total concentration of proteins within the signalling network increases, so does the sharing of phosphoryl groups. As a result, for increasing total protein concentration the overshoot amplitude increases independently of the external ligand concentration. However, as the amplitude increases, the adaptation time decreases, thus decreasing the cell response time.

Numerous theoretical studies of *E. coli* chemotaxis signalling have failed to observe overshoot (for example Clausznitzer et al. 2010; Tu et al. 2008; Likpow 2006; Morton-Firth 1999). There are likely to be numerous different reasons for this. Firstly, it is common in establishing a parameter set for use within a mathematical model, to utilise experimentally determined average protein concentrations. In terms of the average behaviour of a cell population, this would appear to be a reasonable approach; however, it fails to account for the effects of the significant stochastic variation in protein concentrations observed between individual cells (Korobkova et al. 2004), effects that are shown here to be an important determinant of the transient cell response. Secondly, simplified mathematical models give a number of benefits, particularly in terms of the ease with which analytical results may be obtained. However, such simplifications not only alter the network structure, but also the ability of the system to exhibit previously observed transient behaviours, such as those demonstrated here. In particular, the ability of the model to fit with biological observations may be greatly altered or lost altogether. One such example is the model due to Tu et al. (2008) which has been used with some success in a number of studies such as that by Kalinin et al. (2009). However, it has been shown here that within a tenfold variation in the total concentrations of all chemotaxis proteins this particular model does not exhibit oscillatory behaviour.

In the context of previous work regarding Tar/Tsr crosstalk, we believe our work demonstrates CheB-P negative feedback regulation in the context of the signalling cascade is itself enough for the system to exhibit oscillatory overshoot dynamics. Whether this, as well as Tar/Tsr crosstalk, is mutually beneficial or exclusive requires further experimental work. The work presented here contains a number of theoretical results that may be investigated in an experimental setting. In particular, the results presented in Figs. 6, 7, 8 and 12 are testable by appealing to certain under/overexpression mutant cells. In the case of Fig. 6, it is possible to create cells under/overexpressing certain proteins. To investigate Fig. 7, the *meche* and *mocha* operons may be under/overexpressed. Finally, Figs. 8 and 12 would require cells under/overexpressing all of the chemotaxis signalling proteins simultaneously. In each case, once under/overexpression mutant cells are available, analysis of flagella rotation time courses during which cells are challenged with a step change in ligand concentration should reveal the amplitude of oscillation exhibited by cells (if any).

Also, demonstrated here is that total protein concentration is an important factor in affecting the temporal response of an intracellular signalling cascade. We believe that the three key ingredients for determining variation within a network response are its structure, its kinetic rates and the total concentrations of its constitutive elements. We would expect these principles to be relevant in explaining similar phenomena within other biological systems. The first two of these aspects are well-founded results within the analysis of signalling cascades (Kollmann et al. 2005). The third allows us to determine how the transient cell response will vary given different starting conditions of the signalling cascade, namely total protein concentration in this example. Results obtained here indicate that the simplification of using population average values may mask the inherent effects of cell-to-cell variability. It would therefore seem sensible, in addition to studying population behaviour using average values, to consider the potential effects of cell-to-cell variation when considering transient behaviour in cellular systems.

Many biological systems exhibit a large degree of individual variability across their populations. This is usually put down to genetic differences, environment and history. However, even cells identical under these criteria display behavioural variability (Raser and O’Shea 2005). This is likely to be caused by the low copy numbers of components including DNA and key regulatory molecules, leading to stochastic effects (Elowitz et al. 2002). A significant stochastic cell-to-cell variation in concentrations of the *E. coli* chemotaxis signalling proteins has regularly been referred to within the literature (Emonet and Cluzel 2008; Korobkova et al. 2004; Levin et al. 1998; Li and Hazelbauer 2004; Park et al. 2010; Spudich and Koshland 1976). It has also been suggested that, when faced with cell-to-cell variation, reliable signal processing systems will be able to maintain key features (Steuer et al. 2011). A number of features, such as precise adaptation, have been shown to be robust in *E. coli* chemotaxis signalling (Alon et al. 1999; Barkai and Leibler 1997). There are, however, numerous consequences associated with this stochastic variation discussed within the literature (Barkai and Leibler 1997). Most commonly studied are the effects of variation in the concentrations of proteins CheB and CheR, those directly involved in adaptation. In studying these effects, it was noted that they have a significant impact on the adaptation times of cells (Emonet and Cluzel 2008), a feature noticeable within results obtained here (Fig. 8). It has also been shown that varying the total signalling protein concentrations can result in different steady-state phosphorylation levels (Levin et al. 1998). This was observed within our work, which suggests that the sharing of phosphoryl groups between CheB and CheY may be important in the occurrence of numerous phenomena, including oscillatory behaviour.

In terms of a wider picture, cell-to-cell variability coupled with the signalling network structure could be vital for population survival, from both an evolutionary perspective as well as in terms of pure survival, especially for those cells subjected to a wide range of environmental conditions (Bitbol and Wingreen 2015; Dufour et al. 2016; Edgington and Tindall 2015; Frankel et al. 2014; Spudich and Koshland 1976; Waite et al. 2016). We believe our work demonstrates that total protein concentration is an important factor in affecting the temporal response of an intracellular signalling cascade. It suggests that three key ingredients are required for determining the individual cellular response: (i) the protein–protein network structure; (ii) the stoichiometry and kinetic rate values; and (iii) the total concentration of the constitutive elements, in this case proteins. We would expect these principles to be relevant in explaining similar phenomena within other biological systems. The first two of these aspects are well-founded results within the analysis of signalling cascades (Kollmann et al. 2005). The third allows us to determine how the transient cellular response will vary given different starting conditions of the signalling cascade, namely protein concentration in this example. Thus, whilst network signalling structure and stoichiometry may be universal to a species, it is the variation in the total protein concentration that infers individuality and response to their environment by defining their *own* equilibrium protein concentrations and *how* they will return to them (e.g. oscillatory, damped or monotonic). This may have evolutionary advantages in that cells can respond differently to external factors, thus ensuring certain members of a population may survive for a given set of conditions, whereas others may not, thereby ensuring the overall survival of the species. Results obtained here indicate that the simplification of using population average values may mask the inherent effects of cell-to-cell variability. It would therefore seem sensible, in addition to studying population behaviour using average values, to consider the potential effects of cell-to-cell variation when considering transient behaviour in cellular systems. However, further work is required to identify the specific benefits (if any) of this phenomenon.
